# The Neuroanatomical Basis of Panic Disorder and Social Phobia in Schizophrenia: A Voxel Based Morphometric Study

**DOI:** 10.1371/journal.pone.0119847

**Published:** 2015-03-16

**Authors:** Marisol Picado, Susanna Carmona, Elseline Hoekzema, Guillem Pailhez, Daniel Bergé, Anna Mané, Jordi Fauquet, Joseph Hilferty, Ana Moreno, Romina Cortizo, Oscar Vilarroya, Antoni Bulbena

**Affiliations:** 1 Grup de Recerca en Neuroimatge, Fundació IMIM, Barcelona, Spain; 2 Institut de Neuropsiquiatria i Addiccions, Parc de Salut Mar, Barcelona, Spain; 3 Departamento de Bioingeniería e Ingeniería Aeroespacial, Universidad Carlos III de Madrid, Madrid, Spain; 4 Departament de Psicobiologia i Metodologia de Ciències de la Salut, Universitat Autònoma de Barcelona, Spain; 5 Departament de Filologia Anglesa i Alemanya, Facultad de Filologia, Universitat de Barcelona, Barcelona, Spain; 6 Fundación para la Investigación y la Docencia Maria Angustias Giménez, Germanes Hospitalàries, Barcelona, Spain; University of Medicine & Dentistry of NJ—New Jersey Medical School, UNITED STATES

## Abstract

**Objective:**

It is known that there is a high prevalence of certain anxiety disorders among schizophrenic patients, especially panic disorder and social phobia. However, the neural underpinnings of the comorbidity of such anxiety disorders and schizophrenia remain unclear. Our study aims to determine the neuroanatomical basis of the co-occurrence of schizophrenia with panic disorder and social phobia.

**Methods:**

Voxel-based morphometry was used in order to examine brain structure and to measure between-group differences, comparing magnetic resonance images of 20 anxious patients, 20 schizophrenic patients, 20 schizophrenic patients with comorbid anxiety, and 20 healthy control subjects.

**Results:**

Compared to the schizophrenic patients, we observed smaller grey-matter volume (GMV) decreases in the dorsolateral prefrontal cortex and precentral gyrus in the schizophrenic-anxiety group. Additionally, the schizophrenic group showed significantly reduced GMV in the dorsolateral prefrontal cortex, precentral gyrus, orbitofrontal cortex, temporal gyrus and angular/inferior parietal gyrus when compared to the control group.

**Conclusions:**

Our findings suggest that the comorbidity of schizophrenia with panic disorder and social phobia might be characterized by specific neuroanatomical and clinical alterations that may be related to maladaptive emotion regulation related to anxiety. Even thought our findings need to be replicated, our study suggests that the identification of neural abnormalities involved in anxiety, schizophrenia and schizophrenia-anxiety may lead to an improved diagnosis and management of these conditions.

## Introduction

It is very common for schizophrenia to display comorbidity with other psychiatric disorders [1]. In clinical practice, overlapping characteristics in mental disorders often present confounds for an exact diagnostic delimitation. A large number of patients do not fit the Diagnostic and Statistical Manual of Mental Disorders (DSM-IV TR) [2] criteria for a specific disorder or they may qualify for multiple disorders (i.e., comorbidity). Specifically, anxiety disorders (ADs), such as panic disorder (PD), agoraphobia (AG), social phobia (SP), and generalized anxiety disorder (GAD) are frequently comorbid with schizophrenia [3,5], and their prevalence is known to be higher in schizophrenic individuals than in the general population. Recent meta-analyses have reported high rates of ADs in samples of schizophrenic populations [3, 4]. A prevalence of 9.8% was reported for PD, 14.9% for SP and 5.4% for AG [5]. Symptoms of anxiety in schizophrenia are associated with cognitive deficits, a greater risk of relapse, poorer quality of life, and an increased risk of suicide [6]. Moreover, anxiety is considered a basic construct in the development of schizophrenia [7]. In fact, the comorbidity of ADs in schizophrenia seems often to be related to delusions and hallucinations (though such disturbances can occur in the absence of ADs) [8]. Despite its clinical relevance, the comorbidity of ADs and schizophrenia is poorly understood. This highlights the need to identify the neurobiological substrate that underlies the co-occurrence of these psychiatric conditions.

Schizophrenia affects many aspects of behavior, thinking, and emotion, and its physiopathology involves multiple brain regions. Differences in GMV have been well documented in patients with schizophrenia as compared with age-matched controls [9–10]. GMV reductions in frontal, temporal, thalamic, and striatal regions are the most reported findings [11]. It has been suggested that GMV changes may not necessarily result from a unitary pathological process. Instead, these changes seem to stem from neurofunctional abnormalities and disrupted connectivity in a distributed network of frontal, temporal, limbic and striatal regions [12]. This is congruent with previous findings concerning structural abnormalities in these brain regions [9, 13, 14].

Like schizophrenia, high anxiety levels can cause profound distress, suffering, and reduced professional and social achievement [15]. Regarding structural brain changes, previous studies observed alterations in several brain regions in PD and social AD. For instance, PD studies have revealed a GMV increase in the left insula [20], as well as smaller GMVs in the bilateral amygdala [16], the left parahippocampal gyrus [17], right dorsal anterior cingulate cortex [18] and the dorsolateral prefrontal cortex (dlPFC) [19]. As for social anxiety disorder (SAD) prior studies observed decreased GMV in bilateral temporal poles and left lateral orbitofrontal cortex in SAD patients in comparison to normal controls. Moreover, SAD patients were also found to have an increased GMV in the left parahippocampal, middle occipital, bilateral supramarginal and angular cortices, as well as the left cerebellum [21]. Recently, another structural study also revealed increased thickness in the right dlPFC and right parietal cortex in SAD patients [22].

There is a lack of studies on how the coexistence of schizophrenia and anxiety affects brain structure in contrast to each condition by itself. Determining the neuroanatomical substrate underpinning the comorbidity of schizophrenia and anxiety should help to improve diagnostics and management of schizophrenia. To this end, we use voxel-based morphometry (VBM) on a sample including patients with schizophrenia, schizophrenia with specific ADs (PD with and without AG and SP), patients with PD (both with and without AG) and normal controls. Our aim is to determine shared-volume alterations among schizophrenia and specific ADs. Specifically, we are interested in GMV differences in patients with schizophrenia and co-occurring PD and/or SP. Additionally, we examined whether changes in GMV are related to schizophrenic and anxiety symptom severity.

## Materials and Methods

### Ethics Statement

The study was approved by the Parc de Salut Mar Barcelona Clinical Research Ethical Committee, in accordance with the Code of Ethics of the World Medical Association (Declaration of Helsinki).

All participants were given a complete description of the MRI and clinical examination before written informed consent was obtained. In order to ensure full capacity to consent, only patients who were in a stable clinical condition were included. Patients with acute psychosis were excluded from the study.

### Participants

The study subjects included 20 schizophrenic (SCZ), 20 anxious (ANX) patients, 20 schizophrenic patients with comorbid anxiety (SCZ/ANX) and 20 healthy controls (CTRL) matched for age, gender and handedness. The ANX group included ten PD patients and ten PD patients with AG. The SCZ/ANX group included nine PD patients, six PD patients with AG and five social-phobic patients.

### Diagnosis and clinical Assessments

Patients were recruited from the Institut de Neuropsiquiatria i Addiccions del Parc de Salut Mar, in Barcelona, Spain. This procedure involved a comprehensive assessment of the medical and psychiatric history of the patients and a structured interview in order to determine whether they fit the inclusion criteria. Diagnosis was performed during a stabilized period and performed by clinical investigators based on the DSM-IV criteria and the Structured Clinical Interview for DSM-IV (SCID) [2]. According to these criteria, patients were placed in: (a) the SCZ group when exclusively fulfilling schizophrenia criteria; (b) the SCZ/ANX group when fulfilling schizophrenia and any diagnosis of anxiety (PD with or without AG or any type of phobia): and (c) into the ANX group when fulfilling diagnostic criteria for PD with or without AG and any type of phobia. Patients with comorbidity for DSM-IV axis I or axis II disorders were not included, with the exception of SCZ/ANX patients.

It is important mention that, for those subjects medicated with antipsychotics at the time of the scan, doses were converted into chlorpromazine-equivalents, as reported in Table 1. Specifically, 35 of the diagnosed schizophrenic patients were treated with typical antipsychotic medication and two were treated with atypical antipsychotic medication. Regarding the panic and social phobic samples, one patient was taking Vandral and eight were treated with benzodiazepine and selective serotonin reuptake inhibitor anti-depressive medication (SSRIs).

**Table 1:** Sociodemographic and Clinical Data.

	SCZ/ANX (n = 20)		SCZ (n = 20)		ANX (n = 20)		CTRL (n = 20)			
	M	SD	M	SD	M	SD	M	SD	*H value* ^*a*^	*P* value
Gender M/F	12/8		11/9		5/15		12/8		6.73	0.081
Age	32.55	6.901	35.9	0.733	30.90	6.639	33.20	6.613	1.44	0.697
PANSS Positive	13.52	5.74	12.25	3.66	NA	NA	NA	NA	0.44	0.505
PANSS Negative	18.52	7.82	15.50	5.29	NA	NA	NA	NA	1.18	0.278
PANSS Total	65.58	21.41	59.35	16.40	NA	NA	NA	NA	0.45	0.500
Medication	239.31	196.12	285.62	187.02	NA	NA	NA	NA	0.13	0.715
K-Bit	95.65	11.03	91.05	13.76	103.65	7.22	114.65	7.11	40.64	**<0.001**
Years of education	8.80	3.302	8.60	3.068	9.75	3.626	13.60	2.873	19.658	**<0.001**
STAI-Trait	32.45	4.31	32.00	8.37	31.35	9.90	11.40	6.82	34.18	**<0.001**
STAI-State	21.30	10.68	16.21	9.78	21.30	10.65	16.60	8.94	3.77	0.286
LSAS	57.63	23.44	31.33	19.17	38.68	22.30	19.95	2.34	24.23	**<0.001**
SASS	32.45	4.31	32.00	8.36	36.68	8.62	39.50	4.45	17.15	**<0.001**
WALDROP	3.00	2.02	4.00	1.86	2.60	2.15	0.16	0.36	29.44	**<0.001**

Abbreviations: GENDER: Male/Female; ANXIETY DIAGNOSE: Panic Disorder/Panic Disorder with Agoraphobia/Agoraphobia /Social Phobia; PANSS: Positive and Negative Syndrome Scale; Medication; Chlorpromazine equivalents from typical and atypical antipsychotics; KBIT: Kaufman Brief Intelligence Test; STAI: State-Trait Anxiety Inventory; LSAS: Liebowitz Social Anxiety Scale; SASS: Social Adaptation Self-evaluation Scale, WALDROP: Waldrop Physical Anomaly Scale.

a. H: Kruskall-Wallis Test All groups comparisons were thresholded at p values <0.05.

The evaluation of disease severity and psychopathology included the Positive and Negative Syndrome Scale (PANSS), the State-Trait Anxiety Inventory (STAI) [23], the Liebowitz Social Anxiety Scale (LSAS) [24], and the Waldrop Physical Anomaly Scale (WALDROP) [25]. Examination of all subjects also included other measures: the Kaufman Brief Intelligence Test (K-BIT) [26] and the Social Adaptation Self-evaluation Scale (SASS) (see Table 1).

### MRI acquisition

In order to measure between-group anatomical differences, MRI data sets were acquired on a 1.5 T General Electrics (GE) scanner and high resolution T1-weighted 3D-MPRAGE were acquired for each participant (TR, 11.6 ms; TE, 4.9 ms; field of view, 230 mm; matrix, 256 × 256; 104 contiguous axial slices; voxel size, 1.02 × 1.02 × 2 mm). Tissue volume (grey matter) measures were performed using the SPM5 software (Welcome Department of Imaging Neuroscience, London; http://www.fil.ion.ucl.ac.uk/spm) running on Matlab 7.0 (Math-Works, Natick, MA, USA). We used SPM5 for the imaging analysis due to the fact that, even though SPM8 is available since 2009; we started this study before, and therefore, several steps of the preprocessing were performed using SPM5. Finally, every scan was checked for image artifacts and gross anatomical abnormalities.

### Statistical Analysis

Sociodemographic and clinical data were analyzed with SPSS 21 (SPSS for Windows Rel 21 SPSS Inc, Chicago IL). A non-parametric test, Kruskall-Wallis, was used to test for differences between the studied groups, given that our sample did not show a normal distribution. The results of the sociodemographic and clinical data are shown in Table 1, as well as the statistics of the performed distribution test.

For the MRI analysis, we applied standard procedures implemented in the VBM5 toolbox, which extends the new unified segmentation approach implemented in SPM5 [27]. The unified segmentation provides a generative model of VBM preprocessing in which tissue classification, bias correction, and image registration are integrated within the same model. Each reoriented image was segmented and then the final tissue maps of the GM, white matter, and cerebrospinal fluid were modulated with the deformation fields obtained by normalization to standard space in order to analyze volumetric differences between groups. For this study, only the GMV maps were used for statistical analyses. Finally, the modulated grey-matter partitions were smoothed with a 12mm FWHM (Full-Width Half Maximum) Gaussian Kernel and then entered into statistical analyses.

The general linear model was used to estimate differences in GMV by performing a two-by-two between-subjects factorial design in order to analyze the effect of anxiety and schizophrenia, as well as the interaction between factors. All analyses were performed with a threshold of *p* < 0.001 with an extended threshold of 20 voxels.

We performed an interaction-contrast analysis in order to determine if there were GMV differences related to the comorbidity of schizophrenia and anxiety. Subsequently, we constructed the linear contrasts to determine volumetric differences among those groups. In order to discern if these differences were due to the interaction between schizophrenia and anxiety, we applied the interaction contrast as an inclusive mask to the contrasts of interest (e.g. the “SCZ/ANX > SCZ” and “SCZ/ANX < SCZ” contrasts solely).

We also performed the linear contrasts “SCZ/ANX > ANX” and “SCZ/ANX < ANX.” Additionally, the SCZ and the SCZ/ANX groups were compared to the CTRL group. Finally, we also compared the ANX group with the SCZ and the CTRL groups. In order to further elucidate our results, we performed correlation analyses between GMV and the clinical data. To this end, linear regression modeling with grey matter probability at each voxel was the dependent variable, and scores of clinical rating scales were the independent variables. LSAS and PANSS negative clinical rating scales were chosen since they are known for being the most frequently correlated scales with biological markers in the literature.

## Results

There were significant differences between groups on several of the clinical ratings (Table 1).

On the PANSS Positive Symptoms Subscale, we observed increased positive symptomatology in the SCZ/ANX group as compared to the SCZ group. Furthermore, the SCZ/ANX group obtained slightly elevated scores on the PANSS Negative Symptoms Subscale, even though not significantly different, as shown in Table 1. The SCZ/ANX group also obtained a higher score as compared to the SCZ group on the LSAS, although not significant (*p* = 0.94). Finally, it is important to mention that we did not observed significant differences between the SCZ/ANX and the ANX patients in the LSAS scores(*p* = 0.629).

Education level and the years of education were also significantly different among groups. However, such differences were not found between the SCZ and SCZ/ANX groups (education level in years: *p* = 1.000). Specifically, significant differences in years of education were found between the SCQ/ANX group and the CTRL group (*p* = 0.001) and between the SCZ and the CTRL group (*p* = 0.001). Significant differences were also found in the K-BIT scores, as an index of IQ, as indicated in Table 1. However, this variable did not show differences between SCZ and SCZ/ANX groups (*p* = 1.00). Finally, gender was included as nuisance variable in the imaging analysis, even though it did not revealed significant differences among groups.

**Fig 1:**
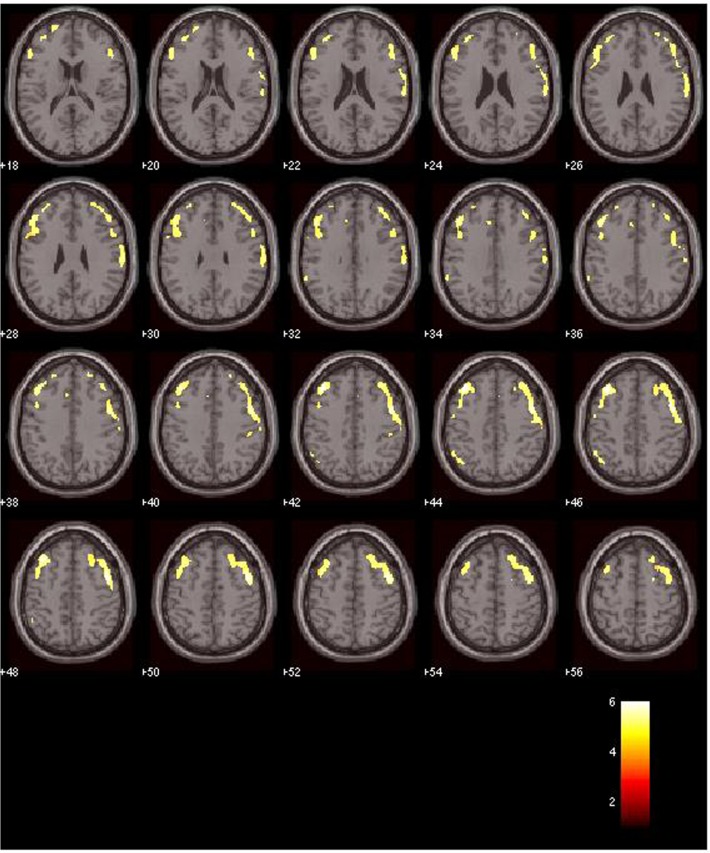
Whole-brain maps illustrate GMV differences in the SCZ/ANX group as compared to the SCZ group. For visualization purposes, the statistical maps were thresholded at P< 0.03-uncorrected. Size and location of clusters are reported in Table 2.

**Fig 2:**
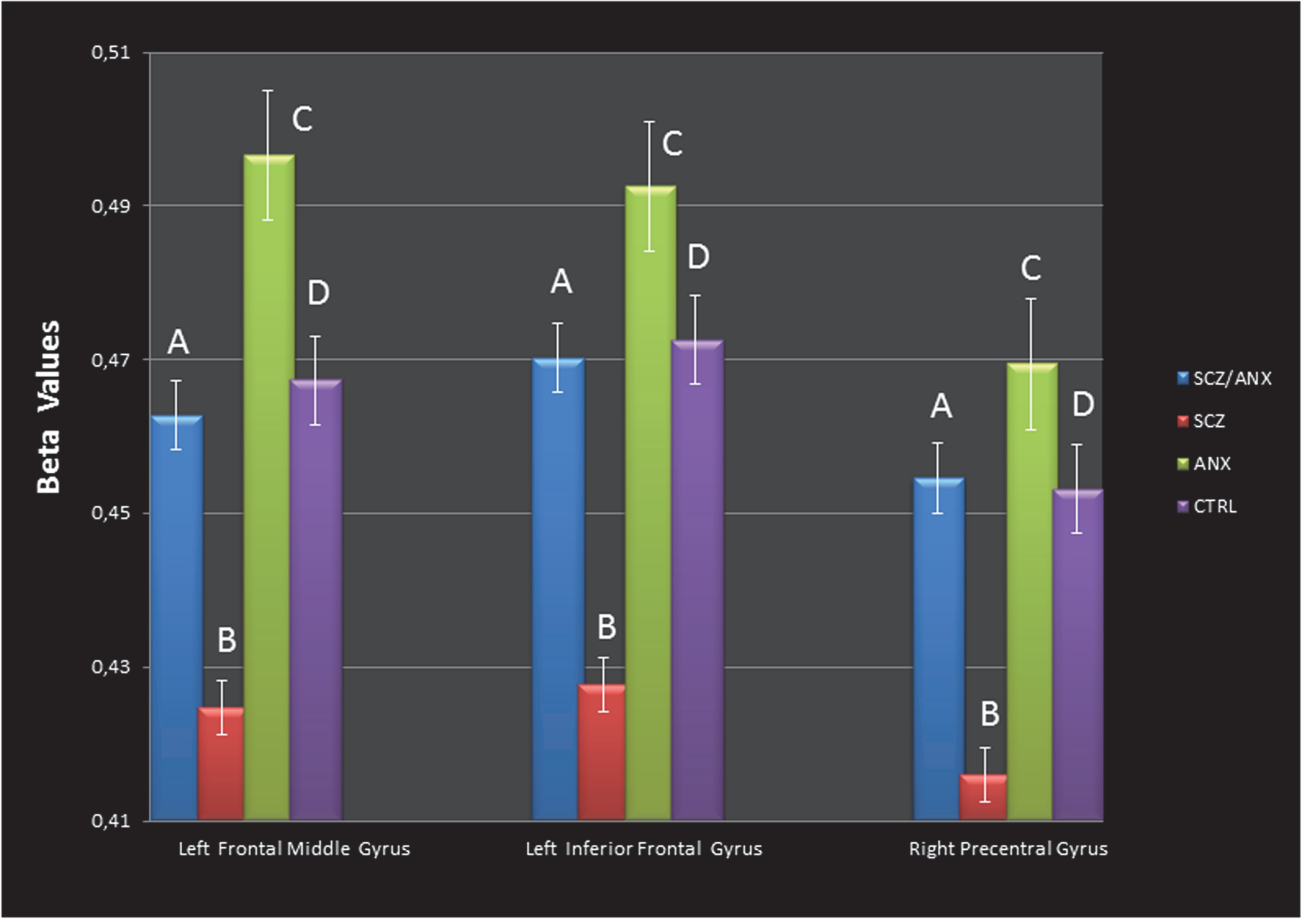
Fig. 2 shows the beta values (Parameters’ extraction) for each ROI that showed GMV differences in the main contrast. a) For the left middle frontal gyrus, significant differences (*p*<0.05) were observed between A-B, A-C, B-D, and B-C. b) For the left inferior frontal gyrus, significant differences (*p*<0.05) were observed between A-B, B-C, and B-D. c) For the right precentral gyrus, significant differences (*p*<0.05) were observed between A-B, B-C, and B-D.

As for the MRI analysis, the results of the interaction analysis (schizophrenia × anxiety) showed GMV differences in the right cerebellum, the left middle frontal gyrus and the left anterior cingulate cortex (ACC), as depicted in Table 2. Furthermore, we observed decreased GMV in the SCZ group as compared to the SCZ/ANX group in the right precentral, right middle frontal and left middle frontal gyri. In order to determine whether the observed volume differences are due to the interaction of schizophrenia and anxiety, the interaction contrast (schizophrenia × anxiety) was applied as an inclusive mask to this contrast (see Table 2, Fig. 1). Moreover, in order to detect more specific GMV patterns in the fore-mentioned brain regions, we obtained the beta values (parameters of GMV) using the whole regions of interest that resulted from the main contrast. Using these values, we performed an ANOVA and found several differences between groups, as shown in Table 3 and Fig. 2. Specifically, significant differences were found between SCZ/ANX and SCZ in the three ROIs. The SCZ/ANX also showed significant differences in the left middle frontal gyrus as compared to the ANX group. Additionally, the SCZ group also showed significant differences in comparison to the ANX and CTRL groups in all the ROIs under study. Finally, although the results did not reach significance, it is worth noting that the ANX group showed higher betas values in the left middle frontal gyrus than the CTRL group.

**Table 2:** Whole-brain analysis.

Brain Region	Center of mass (MIN coordinates)	Cluster size (mm^3^)	*t*	*P* value
**Interaction Contrast**				
Left Middle Frontal Gyrus (BA9)	−37 32 45	257	4.53	<0.001
Left Anterior Cingulate Cortex (BA32)	−9 29 32	75	4.10	<0.001
Right cerebellum	9 −43 −43	258	4.50	<0.001
**SCZ<SCZ/ANX**				
Right Precentral Gyrus (BA6)	46 4 49	1357	5.90	0.003*
	49 2 41		5.33	
Right Middle Frontal (BA9)	45 10 43		5.52	
Left Middle Frontal Gyrus (BA9)	−38 31 45	705	6.44	0.032*
**SCZ<CTRL**				
Left Middle Frontal Gyrus (BA9/45)	−38 31 43	6840	6.44	<0.001*
	−44 29 35		5.30	
Inferior Frontal Gyrus (BA45)	−48 31 27		5.37	
Right Precentral Gyrus (BA44)	45 4 49	126180	5.90	<0.001*
	49 2 41		5.33	
Right Middle Frontal Gyrus (BA44)	34 10 43		5.52	
Left Orbitofrontal Gyrus (BA/10/47/46)	−35 58 0	4798	5.60	<0.001*
	−35 57–10		5.29	
	−21 50 17		5.33	
Left Middle Temporal Gyrus (BA 37)	−49–67 7	1010	5.35	0.010*
Left Angular Gyrus (BA10)	−34 59 2	76	5.42	0.039*

This table shows the results of the whole-brain analysis. Abbreviations: BA: Brodmann area; *T*: statistical value; *P*: significance Value.

* *p* < 0.05 FWE corrected. The contrasts in the opposite direction did not render significant results at this threshold.

**Table 3:** Beta values (Parameters of the regions of interest).

	SCA/ANX		SCZ		ANX		CTRL		
	M	SD	M	SD	M	SD	M	SD	*P* Value
**Frontal Middle Left**	0.462760	0.0484121	0.426520	0.0543054	0.496650	0.0562658	0.467330	0.0425075	*p <*0.001
**Frontal Inferior Left**	0.470270	0.0342436	0.427750	0.0305266	0.492585	0.0386499	0.472570	0.0347582	*p<*0.000
**Precentral Rigth**	0.454550	0.0349078	0.416060	0.0342688	0.469455	0.0371103	0.453175	0.0319953	*p<*0.000

This table shows the results of the analysis performed with the extracted beta values for each Roi of interest.

Abbreviations. M: mean, SD: standard deviation.

Additionally, in order to further elucidate our results, we also performed the contrast SCZ < CTRL and ANX>CTRL, with the aim to determine GMV differences among those groups. There was a decrease in GMV in the SCZ group in frontal, temporal and parietal regions as compared to the controls (See Table 2). The contrast SCZ > CTRL did not reach significance. Additionally, by comparing the ANX and the CTRL groups, we observed that the ANX patients showed GMV increases in the left cuneus and the left inferior frontal cortex (MNI x-42 y-23 z-12, *p* < 0.001, t = 3.62, 59 mm). The ANX group also showed GMV reduction in the right temporal superior and inferior gyri (MNI x63 y-27 z9, *p* < 0.001, t = 3.84, 207 mm, MNI x48 y-12 z-33, *p* < 0.001, t = 3.60, 54 mm), the left occipital gyrus (MNI x-26 y-88 z19, *p* < 0.001, t = 3.53, 24 mm) and the left middle frontal gyrus (MNI x-39 y27 z36, *p* < 0.001, t = 3.32, 21 mm).

Finally, regarding the correlation analyses, the SCZ/ANX group showed a significant negative correlation with the LSAS in the bilateral superior temporal gyrus (MNI x-54 y-42 z18, *p* < 0.001, t = 5.65, 84 mm3; MNI x53 y-46 z8, *p* < 0.001, t = 5.43, 35 mm). In addition, we also observed a negative correlation between the PANSS Negative Symptoms Subscale in this group and GMV in the left Heschl’s gyrus (MNI x-43 y-24 z-11, *p* < 0.001, t = 7.16, 169 mm), right superior temporal gyrus (x57 y-22 z-7, *p* < 0.001, t = 6.92, 259 mm), left superior frontal gyrus (MNI x-24 y57 z2, *p* < 0.001, t = 5.98, 40 mm), left inferior occipital gyrus (MNI x-46 y-61 z-10, *p* < 0.001, t = 5.84, 80 mm) and right inferior temporal gyrus (MNI x56 y-29 z-25, *p* < 0.001, t = 5.0, 22 mm).

## Discussion

We performed a VBM study in order to determine the neural substrate that might underlie the comorbidity of schizophrenia with anxiety. Our study identified GMV decreases in the SCZ group as compared to the SCZ/ANX group mainly in the dorsolateral prefrontal cortex (dlPFC), including the bilateral middle frontal gyrus and the right precentral gyrus. We applied the interaction contrast as an inclusive mask to this comparison, in order to ensure that our results are related to the co-occurrence of both disorders. Additionally, the extracted GMV parameters of these regions indicated the possiblity of an anxiety related effect observed in the SCZ/ANX and ANX patients, with GMV increases observed mainly in frontal regions, incliding mostly the DLPFC.

Specifically, this region has been suggested to be responsible for attention and working memory, as well as motor planning, organization and regulation [28, 29]. This region has also been related to cognitive/behavioral aspects such as preventing or/and anticipating [30]. Neurofunctionally, frontoparietal networks, including the dlPFC, ACC and the parietal cortex, have been related to executive, emotion regulation and attentional functions [31, 32]. Thus, the DLPFC has been shown to be implicated in emotion regulation circuits in several studies [33, 34, 35, 36] and is also known to be more active during emotion suppression [37, 38].

A review on structural abnormalities within this area in schizophrenia reported deficits in the left middle frontal gyrus in 50% of the reviewed studies [8]. It has also been suggested that first episode schizophrenic patients present altered cortical thickness mainly in prefronto-temporal regions [39]. All these findings suggest the dlPFC as one of the most implicated regions in schizophrenia. Our study also indicated GMV reductions in frontal areas in the schizophrenic patients, as compared to the CTRL group. Longitudinal studies in chronically schizophrenic patients have also reported a progressive GMV loss that was most pronounced in frontal areas, associated with poor outcome and more negative symptoms [40]. It has been suggested that neurodevelopmental disturbances in schizophrenia might be occurring during the first episode of the illness, and this could indicate that brain alterations are not merely related to the effects of chronicity or medication [41]. GMV reductions in the dlPFC have been found in never-medicated first-episode schizophrenic patients [42] and dysfunction in this region has also been associated with deficits in working memory in these patients as compared to controls [43]. Such findings indicate that structural brain abnormalities and cognitive deficits might be present before the illness onset but afterwards are observed to follow a progressive pattern [44]. Previous functional Magnetic Resonance Imaging (fMRI) studies have consistently observed reduced dlPFC activity during cognitive control tasks in schizophrenia, associated with impaired task performance and behavioural disorganisation irrespective of patient medication status [43, 45–46].

However, none of these findings have controlled for the anxiety component among schizophrenic patients. There is increasing evidence that relates the prefrontal cortex with the pathogenesis of anxiety disorders [46, 47]. Specifically, the dlPFC has been related to cognitive and behavioural aspects strongly relevant for certain aspects of anxiety symptoms such as excessive, pervasive, and uncontrollable feelings of anticipating the worst or danger [30]. This region have shown to be implicated in emotion regulation and/or suppression [33, 34] therefore, deficits in such brain region could be related to anxiety`s symptoms [22].

Keeping with the results observed by Bruhn et al. [22] we found smaller GMV increases in the SCZ/ANX group in bilateral dlPFC as compared to the SCZ group. Moreover, the extracted parameters from these regions indicated higher beta values in the ANX group than in any of the groups, even compared to the CTRL group, though no significant. As for the imaging results, the ANX group also showed GMV increases in frontal regions, which included the right medial OFC, the right ACC, the rigth superior frontal gyrus and the left cuneus, in comparisson to the CTRLs.

Considering our results and previous findings by other studies, we suggest that smaller GMV decrease in the SCZ/ANX group in the dlPFC may be related to maladaptive emotion regulation related to anxiety, resulting in a compensatory GMV increase that might be associated to the comorbidity of both conditions. Our findings are supported mostly by a) the fact that we did not observed these GMV alterations in the SCZ group as compared to the CTRLs, b) the ANX group showed higher GMV parameters in the extracted Rois than any group and also GMV increases in frontal regions as compared to the CTRls and c) prior findings have related compensatory increases in the dlPFC to anxiety symptoms [22, 48]. It has been suggested that a diminished regulation of bottom-up regions (mostly temporal) by prefrontal areas might play a crucial role in anxiety common symptoms [49].

A possible explanation for the relative preservation of GMV in SCZ patients with comorbid anxiety may be related to a compensatory increase of GMV in the dlPFC. Concretely, this region has shown to be implicated in emotion regulation circuits in many studies [33, 34, 35, 36]. Therefore, greater volume in this brain structure might be the result of a continuous effort of emotion regulation. Thus, previous findings have observed increased cortical thickness in social anxiety patients in the dlPFC [22], which supports the results of the current study, even thought that clinically, the SCZ/ANX presents similar deterioration to the SCZ group, as reflected in the applied scales.

As for the results of the interaction contrast, we observed GMV differences in the right cerebellum, the left middle frontal gyrus and the left ACC. Such GMV differences might represent the co-occurrence of schizophrenia and anxiety. As previously mentioned, the left middle frontal gyrus has been found to present structural abnormalities in schizophrenia, associated with cognitive and attention-related impairments. This region has been related with emotion regulation [42, 43, 45, 50] and it has thought to be implicated in anxiety’s physiopathology. The ACC has also been related to attention/emotion modulation [51] and has been implicated in social anxiety disorder [48]. Prior structural studies have indicated volumetric alterations in specific networks have also been observed, including the dorsal prefrontal cortex, ACC, right parahippocampal gyrus, thalamus, cerebellum, and pons, in relation with executive dysfunction in patients with schizophrenia [52]. However, further research is needed in order to determine the possible neuranatomical effects on the cerebellum that the comorbidity of anxiety and schizophrenia might implicate.

Interestingly, the ANX group showed increased GMV in the right medial OFC, the right ACC, the right superior frontal gyrus and the left cuneus, as compared to the controls. As mentioned before, the ACC and the OFC have been associated with social anxiety disorders [49]. As for the superior frontal gyrus, fMRI studies have observed that the superior frontal gyrus may be involved in self-awareness, in coordination with the sensory system [53]. Such processes might be relevant as part of anxiety symptomatology. Further research is needed in ordert to corroborate the nature of the cuneus’s involvement in the pathophysiology of anxiety, though there is evidence pointing to an abnormal processing of emotionally neutral faces and places in patients with panic disorder [54].

Finally, our study revealed extensive structural alterations in the SCZ patients as compared to the CTRLs. Our findings are consistent with previous studies, which implicate frontal, temporal and parietal regions, as those most frequently showing GMV reductions in the SZC patients. These findings are also consistent with the suggested conception that schizophrenia might arise from dysfunction of fronto-temporal circuits [55].

Taken together, our findings suggest that GMV in these brain regions may account for alterations related to interaction that underpins the comorbidity of both conditions and the associated symptoms. Our results are in consonance with those found by Bruhl et al. [22] and might point to a distinct clinical and neuroanatomical basis in schizophrenic patients with comorbid anxiety. However, the hypothesis that compensatory GMV increases in the dlPFC in association with anxiety’s abnormal emotion suppression might be related to smaller GMV decreases in the dlPFC in schizophrenic patients with comorbid anxiety requires further investigation.

We also observed important correlations between GMV and the clinical data. Our findings indicated a negative correlation between the SCZ/ANX group and the social anxiety scale (LSAS) in the bilateral superior temporal gyri. The superior temporal gyrus has shown to be an important structure in social cognition processes [56] and temporal lobe alterations have also been found in PD [57]. Therefore, deficits in this brain region might be related to difficulties to adequately process relevant social stimuli, such as the perception of emotions in facial stimuli, since this structure has been observed to play an important role in the perception of emotions in facial stimuli. Interestingly, previous clinical studies have related clinically meaningful anxiety with schizophrenia, and, specifically, with poorer psychosocial function [58]. Additionally, we also observed a negative correlation the PANSS Negative Symptoms Subscale and left Heschl`s gyrus, right superior temporal gyrus, left superior frontal gyrus, left inferior occipital gyrus and right inferior temporal gyrus. In line with prior literature, these findings suggest that structural abnormalities in temporal cortices may contribute to the pathophysiology of schizophrenia [59, 60] since progressive volume reduction has been reported in the temporal lobe [61] as well as Heschl's gyrus/planum temporale gray matter [62]. It is worthwhile to point out that abnormalities in the temporal lobe have also been related to negative symptomatology [60]. Furthermore, structural studies in social anxiety disorder have also observed anatomical differences in temporal brain regions [63, 64] suggesting that temporal brain areas might be affected by the comorbidity effects of both disorders. In conclusion, the results of the performed regression analyses seem to the indocate that, by interacting with anxiety, schizophrenia might present specific neuroanatomical differences.

Finally, a number of limitations need to be considered. The progression of the disease and the patients’ medication status might be confounding factors, even though imaging studies have shown brain abnormalities in patients with schizophrenia at different stages of the illness [65]. Furthermore, a recent report showed no significant contribution of typical and/or atypical antipsychotics to grey-matter reductions in schizophrenic patients compared to healthy controls [41]. Another limitation of this study is that, although previous studies using schizophrenic patients have used samples with similar differences, our SCZ patients had lower education level and IQs compared to the healthy controls [66]. This cognitive impairment often pre-dates the illness onset and it might represent an intrinsic part of the illness and it is observed in young, drug-naïve patients [67].The distribution of the anxiety group might also be a limitation in our study, since it included both PD (with and without Agoraphobia) and SAD diagnoses. Additionally, another limitation is our relatively small sample size. It therefore would be important to replicate our findings in bigger samples in order to confirm our results.

Overall, considering the results reported herein, our findings seem to reveal a possible neuroanatomical substrate that could account for schizophrenia and specific anxiety disorders. Our findings might help improve current diagnoses and treatment of schizophrenic patients, with better outcome predictors and pharmacological selection. However, additional studies in bigger samples and newly diagnosed antipsychotic-naive patients with anxiety are needed to replicate preliminary findings and further investigate these associations.
