# Correction: The Neuroanatomical Basis of Panic Disorder and Social Phobia in Schizophrenia: A Voxel Based Morphometric Study

**DOI:** 10.1371/journal.pone.0126407

**Published:** 2015-04-23

**Authors:** Marisol Picado, Susanna Carmona, Elseline Hoekzema, Guillem Pailhez, Daniel Bergé, Anna Mané, Jordi Fauquet, Joseph Hilferty, Ana Moreno, Romina Cortizo, Oscar Vilarroya, Antoni Bulbena

The following information is missing from the Funding section: PI11/01433—FEDER.

The complete, correct funding information is as follows: This work was supported by grants from Instituto de Salud Carlos III-FEDER, (PI11/01433). MP was supported by a FI grant of the Agencia de Gestió d’Ajuts Universitaris i de Recerca. The funders had no role in study design, data collection and analysis, decision to publish, or preparation of the manuscript.
